# Teaching-Learning Processes: Application of Educational Psychodrama in the University Setting

**DOI:** 10.3390/ijerph17113922

**Published:** 2020-06-01

**Authors:** Jesús Maya, Jesús Maraver

**Affiliations:** 1 Department of Psychology, Universidad Loyola Andalucía, Avda. de las Universidades s/n, Dos Hermanas, 41704 Seville, Spain; 2 Department of Developmental and Educational Psychology, University of Seville, Camilo José Cela s/n, 41018 Seville, Spain; jesmargil@alum.us.es

**Keywords:** teaching-learning process, psychodrama, role-playing, higher education, teaching innovation, teaching needs, teaching effectiveness

## Abstract

The use of effective teaching strategies should be developed from teachers’ reflections on educational needs. This study has a twofold objective: to identify needs in teaching-learning processes in the university setting as well as to present and examine the effectiveness of four psychodramatic techniques: psychodramatic images, soliloquy, role-playing and *roda viva*. A qualitative design using thematic analysis was followed. All 128 teachers participating in the Training in Teaching Skills: Educational Psychodrama (nine courses) were evaluated. Teachers (62.5% women) were from different disciplines. Two semi-structured group interviews were conducted using the focus group procedure. Focus groups were held at the beginning and end of each course (18 in total). The phases of thematic analysis were used as discourse analysis strategies. Teachers reported the need to develop active teaching practices with large groups, strategies to motivate students and skills for conflict resolution with students. Concerning psychodramatic techniques, emphasis was placed on the psychodramatic images to promote active learning and group construction of contents, exploring previous ideas and as an evaluation resource. In addition, the structured use of role-playing was positively assessed. These results identify specific teaching needs and support the use of psychodramatic techniques as a valuable educational resource in higher education.

## 1. Introduction

Achieving teaching excellence in university education is one of the top priorities in current university teaching plans [1,2]. The definition of teaching excellence has been conceptualized from a variety of approaches. Currently, there is considerable controversy about what makes an excellent teacher in higher education [3]. Despite the complexity of the term, teachers perceive teaching excellence in relation to teaching practices that lead to significant student learning [3]. In fact, present-day teaching-learning models focus on meaningful learning of content [4]. These models require teachers to acquire specific skills and specialize in teaching practices that are alternatives to the traditional approach [5,6]. To do this, universities must address new teaching needs. Accordingly, higher education must promote a series of programs that guarantee the ongoing training of teachers to achieve teaching excellence [7,8]. The use of evidence-based, effective, up-to-date and innovative teaching methods by teachers is considered particularly important in present-day universities [7,9].

Teaching-learning processes should consider those variables that predict good student performance. Currently, teaching style and teaching practices are considered relevant dimensions for academic performance [3,10]. Systematic reviews confirm a strong association between teaching methods and student performance [11]. Teaching practices such as clear goal setting and ongoing teacher feedback have been recognized as evidence-informed practices to optimize academic outcomes [11]. Additionally, other variables such as the specific content of the curriculum, student profile or student motivation can also influence academic results [7,10]. Although a teaching style predominates (for example, a more traditional teaching style focused on knowledge transmission as opposed to a more constructionist style focused on students discovering knowledge), teacher-student interaction is a dynamic process, in which the teacher must continuously adapt to educational, institutional and contextual changes specific to each university and to social and political changes outside the university [12].

In this evolving situation of teaching in higher education, one of the current challenges of Educational Psychology is to develop and implement evidence-based teaching practices in various teaching-learning situations [3,7]. One of the first steps towards achieving effective teaching-learning processes is the development of a teacher needs evaluation. Several authors note the importance of seeking teaching excellence based on the perceived needs of teachers through analysis and reflection on their own teaching practice [1,9]. Teacher training programs recommend developing cognitive processes of reflection on one’s own teaching practice and strategies [8]. Therefore, university teacher training programs focused on achieving teaching effectiveness should be implemented according to teaching experience in a specific learning situation in current higher education.

Teaching effectiveness has been widely studied in teaching-learning processes for several decades [13,14]. Teaching effectiveness can be defined in terms of five components: personal traits of the teacher, teaching styles, classroom management, mastery of teaching content and the ability to anticipate and make appropriate decisions [14]. Current studies provide new evidence on the influence of different variables. Dimensions such as the leadership exercised by the teacher, emotional intelligence, the emotion experienced by the teacher during teaching, experience or the teacher’s ability to resolve conflicts in the classroom seem to influence the teaching effectiveness of university teachers [15,16,17]. Consequently, teaching effectiveness appears to be influenced by a set of interrelated variables. However, teaching style and teaching strategies have been the dimensions most studied in predicting teaching effectiveness [18,19,20].

Recently, there has been an increase in teacher training courses focused on innovative methodology in higher education to achieve teaching effectiveness. These methodologies focus on experiential learning and the shared construction of knowledge between students and teachers. Flipped room, problem-based learning or project-based learning are some of the current methodologies used in universities [3,7,21,22]. These methodologies share the importance of giving students an active role in the construction of knowledge in the teaching-learning process, while the teacher acts as a facilitator of learning [5,7,21]. However, some teachers may find it difficult to implement active, experiential and dynamic learning practices as alternatives to the traditional educational approach focused on the transmission of knowledge. These difficulties may be due to the lack of pedagogical training of teachers in different disciplines, the density of the contents of the teaching plans, the difficulties of balancing teaching and research or overcrowding in the classrooms [23].

This study proposes and presents a series of educational practices based on the reflection of teachers on their teaching practices, on the existing need to improve as teachers and on the obstacles encountered in the modern university.

### 1.1. Educational Psychodrama in the University Setting

Currently, psychodrama is recognized by different governments including Austria and Hungary as an effective approach to promote healthy behaviors [24]. In recent years, systematic reviews in psychodrama have increased, focusing on two objectives: (a) the definition and review of psychodramatic techniques, and (b) the analysis of the effectiveness of psychodramatic interventions in the clinical context [24,25]. This study aims to contribute to expanding the evidence on psychodramatic techniques applied to the educational context. The origins of educational psychodrama are in the group psychodrama proposed by Jacob Levy Moreno [26]. This author identified the educational context as suitable for the application of psychodrama. The educational setting is considered a field of application in which psychodrama can be used as a theoretical-practical model to improve learning processes [26]. Specifically, the application of psychodramatic techniques enhances creativity in teaching-learning processes. Similarly, psychodrama focuses on the use of action to promote effective learning [26,27].

On a theoretical level, educational psychodrama shares with constructionist paradigms the notion of students as active agents in their learning process to achieve the meaning of the contents. Educational psychodrama or pedagogical role-playing is defined as a teaching-learning process based on the integration of personal interrelationships, action, the use of games and active teaching techniques [27,28]. The application of these psychodramatic elements in higher education has also been termed psychodrama-based training [29]. From this perspective, the use of psychodramatic techniques in the educational context is used to optimize learning processes [29]. Furthermore, in educational psychodrama, the emotional and experiential component of learning takes on special relevance [27]. On the one hand, some studies show the importance of emotion as an enabling dimension for creativity, knowledge and skill acquisition in learning processes [4,30,31]. On the other hand, studies from different disciplines, such as psychology or business administration, show that experiential learning methods enhance the meaningful learning of the contents [4,32,33]. In this sense, psychodrama considers three stages in the learning process and in the assimilation of knowledge: memory, play and dramatization. These stages coincide with the process that students carry out to register, elaborate and implement information mediated by attention processes [28].

In recent years, the use of psychodrama in formal education has been increasing. Educational psychodrama has been implemented mainly in high schools [28,34,35]. In this setting, the application of psychodrama appears to promote knowledge acquisition, abstract thinking and socio-affective processes [35,36,37]. Concerning the acquisition of knowledge, the literature shows the relevance of psychodrama for learning content in different disciplines, such as history and languages, as well as for the professional specialization of students [28,36]. There is less evidence of the application of psychodrama in higher education. However, use of psychodrama in the university shows positive results for knowledge acquisition and professional role training [29,38,39]. Specifically, evidence shows the positive effects of psychodramatic techniques on the acquisition of professional skills in education, psychology and business students [27,38,39]. This empirical evidence justifies the need to develop a systematization of the educational psychodrama applied in universities.

Psychodramatic techniques such as role-playing, role reversal, *roda viva*, mirror, psychodramatic images or soliloquy are used in educational psychodrama [27,28]. Techniques such as role-playing are frequently used in the university setting, although systematic reviews of their efficacy are limited to the clinical setting [24,40]. Psychodramatic techniques are characterized as active techniques through which the protagonist, in this case students, can learn by exploring, deepening and constructing academic content with the support of practical, visual and kinesthetic elements [28]. Role-playing enables students to put content into practice, psychodramatic images enable exploration of the possibilities of a content and soliloquy facilitates the verbalization and expression of emotions and contents [27,28]. However, the use of these techniques must be defined and structured in the specific teaching context of higher education.

In summary, although there have been theoretical and practical contributions from educational psychodrama for decades, insufficient empirical evidence has been found on the effectiveness of psychodrama in higher education. Therefore, although in disciplines such as psychology and education, teachers commonly use psychodramatic techniques, we lack systematicity in their application and rigorous results on their usefulness.

### 1.2. Present Study

This study introduces a strategic approach to teaching innovation: Training in Teaching Skills: Educational Psychodrama (TTS-EP). TTS-EP aims to analyze the current needs of teachers and to instruct university faculty in the educational use of psychodrama in the university setting. Specifically, teachers are trained in four psychodramatic techniques: psychodramatic images, soliloquy, role-playing and *roda viva*. The use of these techniques is presented as an educational resource proposal integrated into current teaching needs. Thus, this study seeks to give a voice to teachers as those responsible for teaching processes. The purpose of this study is to promote teacher excellence in the teaching-learning processes based on a current analysis of educational strategies and teaching needs.

The aim of this study is twofold: (1) to identify teachers’ needs in relation to teacher performance and to the difficulties perceived in the teaching-learning processes, and (2) to present and understand the effectiveness perceived by teachers of the different psychodramatic techniques: psychodramatic images, soliloquy, role-playing and *roda viva*.

## 2. Materials and Methods

### 2.1. Study Desgin

The sample comprises participants in the nine TTS-EP courses implemented by the University of Seville between 2017 and 2019. All the teachers who participated in each course were involved in the study. Specifically, a qualitative design is followed by a thematic analysis method, focusing on its constructionist function in which meanings and experiences emerge in a set of discourses [41]. The evaluation follows an internal process carried out by the groups’ implementers in two stages: before the beginning and at the end of the course. In both evaluation periods, different objectives are evaluated independently. In the evaluation at the beginning of each TTS-EP the teaching needs are studied, while in the final evaluation the usefulness of psychodramatic techniques in higher education is evaluated. For each objective, one semi-structured group interview with open-ended questions was developed: an interview on teaching needs in higher education and an interview on the effectiveness of psychodramatic techniques. Accordingly, the data collection procedure followed the quality criteria associated with the implementation of focus groups [42]. Similarly, the qualitative and inductive analysis of the data followed the phases and recommendations of thematic discourse analysis [41]. The data from this study represent the first step in analyzing the usefulness of psychodramatic techniques and establishing evidence-based teaching practices in universities using a psychodramatic approach.

### 2.2. Training in Teaching Skills: Educational Psychodrama (TTS-EP)

TTS-EP is a university teacher training course intended as a proposal for teaching innovation focused on addressing current teaching needs in order to develop evidence-based practices. Specifically, this group training aims to increase knowledge and train teachers in the use of psychodramatic techniques to achieve excellence in teaching-learning processes. The TTS-EP course was first implemented in 2017. The main theoretical components of this psychodrama-based course and the initial findings have been presented in international congresses on education and teaching innovation [43]. This course is based on the role theory of the psychodramatic approach [26,28]. According to this theory, success in the social, family or professional context is mediated by training for a specific role. In this case, to develop the role and improve teaching efficacy, the training of different teaching practices is recommended. Specifically, the training in the use of psychodramatic techniques focuses on strictly educational contents and teaching-learning processes, excluding family events and personal situations of the participants.

**Figure 1:**
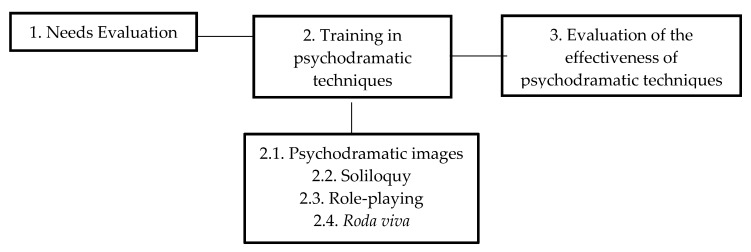
Structure of Training in Teaching Skills: Educational Psychodrama.

Concerning methodology, TTS-EP is carried out during a five-hour intensive course. The training is led by two psychologists specialized in educational psychology and psychodrama. Implementation of TTS-EP involves three different phases. Figure 1 illustrates the course structure.

1. Needs evaluation. This first phase lasts one hour. In this phase, a focus group is conducted with the teachers to explore their teaching needs and challenges. The aim of this phase is to evaluate the opinions and understand the current teaching needs perceived by teachers in the university setting. Specifically, teachers are asked about five contents: teaching effectiveness, needs related to teaching practices, needs in teacher-student interaction, recent difficulties and challenges in higher education and other contextual elements that influence their teaching performance.

2. Training in psychodramatic techniques. This phase lasts three hours and has a dual objective: to define the educational applications of psychodramatic techniques and to train the teachers to apply these techniques in the specific contents taught by each teacher. This phase can be structured in three sub-phases.

Sub-phase 2a: oral presentation of the techniques. First, psychologists give an oral presentation of the four psychodramatic techniques. Specifically, the psychodramatic techniques are defined and their possible variations in higher education are indicated (see Table 1). Then, the trainers show an example of each technique with an academic content taught to psychology students (30 min).

Sub-phase 2b: technique-content association. Individually, each teacher participating in the TTS-EP must reflect on and select content from their academic curricula for each of the techniques; that is, teachers must choose content that can be taught through psychodramatic images, content to be taught through soliloquy, content to use with role-playing and content to be worked with applying *roda viva*. Afterwards, each teacher tells the training group the selected technique-content association of their curricula. The aim is to generate different possibilities in the application of psychodramatic techniques with different content (for example, a developmental psychology teacher communicates that planned role-playing can be a good technique for learning parenting styles or, for example, a didactics teacher tells the group that the *roda viva* can be used to discuss the differences between cooperative and competitive learning) (60 min).

Sub-phase 2c: psychodramatic technique training. Finally, teachers are trained in the use of the techniques for specific content. A volunteer teacher will apply the technique in an experiential process, supervised by the two trainers. At that time, the class situation is recreated. The volunteer teacher takes on the role of an expert teacher for some particular content while the other teachers take on the role of students. Thus, the volunteer teacher briefly explains the academic content and applies the chosen psychodramatic technique (for example, a developmental psychology teacher briefly explains the parenting styles and gives a script for two other teachers to perform a planned role-playing in an authoritative style). At this point, the two psychologists supervise the development of the training and of the recreation of the class situation to improve the usefulness of the psychodramatic technique. This process is repeated with each teacher participating in the TTS-EP who wishes to do so. It is recommended that the teachers who participate in this specific training come from a range of disciplines in order to generate greater variability in the use of the techniques with different contents. This exercise is repeated for the duration of the time allocated to training in psychodramatic techniques (90 min).

3. Evaluation of the effectiveness of the psychodramatic techniques. This is the final phase of the course and lasts one hour. In this phase, a focus group is implemented with all the teachers who have received training in psychodramatic techniques. After being trained in the psychodramatic techniques, teachers evaluate the strengths and weaknesses of each technique and the perceived usefulness for its application in the university setting.

### 2.3. Participants

A total of 128 teachers from the University of Seville participated in the TTS-EP course during the years 2017, 2018 and 2019. Specifically, 80 female teachers (62.5%) and 48 male teachers (37.5%) were included. In total, nine TTS-EP courses were held. The average group size was 14.2 teachers. The training carried out was of a multidisciplinary nature. Concerning the area of knowledge, 37.5% were from the faculty of Social Sciences, predominantly teachers of Psychology and Education; 20.3% were from the faculty of Engineering and Architecture; 18.7% were from the faculty of Arts and Humanities; 16.4% were from the faculty of Health Sciences and 6.2% were teachers of Sciences. Regarding experience as a university professor, 32.8% of the teachers had less than five years of experience at the University, 23.4% had between five and ten years of experience and 25.8% had between 10 and 15 years of experience, while 18% of the participating teachers had taught for more than 15 years. In addition, 56.2% of the teachers had a stable professional position at the University, while 43.7% had temporary contracts. Moreover, for 41.4% of the teachers, it was their first training course, while 58.6% of the teachers had participated in other teacher training courses at the University in different teaching methodologies such as flipped room or problem-based learning.

### 2.4. Instruments

To respond to the two objectives of the study, two semi-structured group interviews with open-ended questions were designed for this research. The purpose of these interviews was to promote interaction between teachers and the group construction of the contents.

Interview on teaching needs in higher education. This interview explored two blocks of content: teaching effectiveness and teaching needs. Teaching effectiveness was evaluated with two open-ended questions: “What does a teacher need to be effective?,” “How would you like to improve as teachers?” The teaching needs were evaluated according to specific needs in teaching practice, needs in teacher-student interaction and other contextual needs that influence the teaching-learning processes such as needs coming from the university as an institution: “What difficulties do you encounter in teaching?,” “What difficulties do you encounter in teacher-student interactions?” and “What are the new challenges in university teaching today?” This group interview lasted approximately one hour.

Interview on the effectiveness of psychodramatic techniques. The teachers responded in groups to open questions about the application of four psychodramatic techniques in higher education: psychodramatic images, soliloquy, role-playing and *roda viva.* Five open-ended questions were asked: “What are the possible uses of psychodramatic techniques in education?,” “How can they influence the teaching-learning processes?,” “What are their strengths?,” “What are their disadvantages?” and “Which specific techniques may be most useful and in which teaching processes?” This group interview lasted approximately one hour.

### 2.5. Procedure

The TTS-EP course is part of the implementation of the Comprehensive Plan for training teachers of excellence, part of the University of Seville’s Teaching Plan. This course received stable funding from the University of Seville for three years for its implementation. All teachers at the University of Seville may register for the different editions of the course. Each TTS-EP course is limited to a maximum of 15 teachers. Teachers register for TTS-EP online. Of the nine editions of the TTS-EP course, two have been held in the faculties of Education Sciences, Economics and Biology, and one in the faculties of Communication, Agricultural Engineering and Labor Sciences.

Teachers participated voluntarily in TTS-EP. At the beginning of the course, the objectives of the program were explained by the leaders. The confidentiality of the data and the importance of respect among the participants and of human rights were guaranteed [44]. This process followed the recommendations of the Declaration of Helsinki. The implementation of TTS-EP was approved and financed by the University of Seville according to the ethical principles of anonymity, responsibility and equality.

In each five-hour TTS-EP course, two focus groups were held: at the beginning of the session (evaluation teaching practice needs) and at the end of the session (evaluation of the effectiveness of psychodramatic techniques). A total of 18 focus groups were conducted. The two psychologists in charge of the TTS-EP coordinated the focus groups. One of the experts took a more active role in leading the group, while the other expert transcribed the teachers’ comments. The focus groups encourage interaction among participants, the adoption of perspectives, the analysis and deepening of the themes and the appearance of emerging content on the topics discussed [42].

Each focus group lasted approximately one hour. The discussion was structured using two interviews with open and general questions to foster interaction between teachers. These interviews were repeated in all the TTS-EP courses. Once the entire TTS-EP course was completed, the researchers performed the data analysis.

### 2.6. Data Analysis

An analysis of the contents that emerged in the focus groups was carried out with ATLAS.ti 7.0 (Scientific Software Development GmbH, Berlin, Germany) qualitative analysis software. The sequential phases of the thematic analysis were used to analyze the participants’ contributions and to identify the main themes [41]. For this, the two researchers proceeded through the following sequential phases to assess the teaching needs and usefulness of the psychodramatic techniques: generation of the first codes, identification of potential themes from the codes, review of the themes and definition of the themes. Following the quality criteria of the qualitative analyses, the intra-evaluator reliability was assessed [45]. Both researchers reviewed each phase of the sequence twice, that is, their proposed codes and themes. In addition, at the end of the first phase (code generation) and the second phase (identification of potential themes), a peer review was undertaken to compare the results [46]. Three rounds were needed to reach total agreement between the researchers. Once an agreement had been reached and the main themes had been jointly defined between the evaluators, the main results were reported, following the recommendations for drafting the results of a thematic analysis and addressing those themes identified in at least one third of the groups [41]. In addition, the quality criteria for the qualitative approach were followed during the process of data analysis and drafting of results. These include the importance of rigor in theoretical constructions, the importance of sincerity and transparency about possible biases and the importance of presenting significant contributions to society, which in this case comprise advances in teaching practices in higher education [47].

## 3. Results

The inductive analysis of the results enabled the identification of the main themes relevant to the study objectives. Below is a structured analysis of the results for each objective.

### 3.1. Perceived Needs of University Teachers

**Figure 2:**
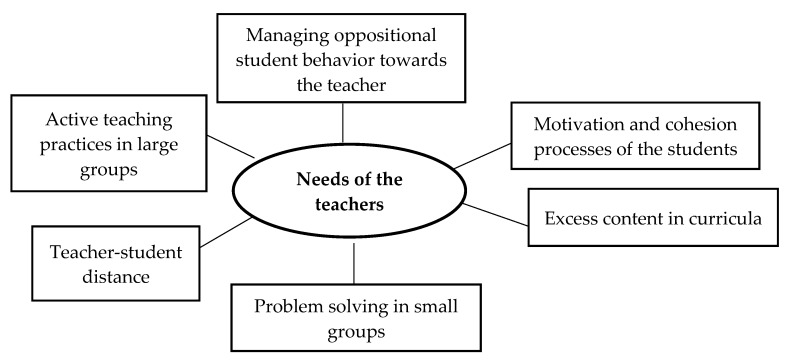
Perceived needs of university teachers.

In the themes that emerged in the focus groups, similar teaching needs were repeated in at least one third of the groups. Figure 2 shows the six current needs identified by teachers in the teaching-learning process in higher education.

Active teaching practices in large groups. The main teaching need expressed was the difficulty of using teaching-learning strategies that differ from the traditional model of theoretical presentation of content. This was particularly true for this type of group (more than 50 students, according to several teachers). In addition, teachers also revealed the difficulty of using active practices when the academic curriculum is rather abstract. Teachers stressed the need for active techniques to manage large groups that encourage active learning through the development and construction of content. An example verbalized by one teacher is: “In small groups I can carry out active and individualized group work and practices, but what should I do with a group of 80 students?”

Teacher-student distance. As a second teaching need, teachers revealed the challenge of managing distance with the student. Distance could be understood as the emotional proximity and warmth between the teacher and the student. Specifically, there were two opposing opinions in the focus groups regarding the consequences of maintaining a greater or lesser distance from the student. To a considerable extent, teachers revealed that in teaching there should be a great deal of emotional proximity to the students. In this close relationship, the teacher becomes a facilitator of learning according to the students’ individual needs. However, these teachers also revealed that in this situation they fear losing their position of authority in the teaching-learning process, in addition to the difficulty of maintaining an equitable position of emotional closeness with all students. To a lesser extent, teachers expressed their understanding that in education there must be distance between teacher and student. Nevertheless, these teachers indicated concern that this distance may be an obstacle to meaningful learning. One teacher stated: “Sometimes I am quite close to the students and I have found that the students have exceeded the limits. So, I wonder if I’m wrong to take such an interest in them.”

Problem solving in small groups. One of the most mentioned teaching needs in the focus groups was conflict management among students in small work groups. Teachers stated that they usually assigned work in small groups (4–6 students). According to the teachers, in these groups, problems appeared among members due to their different levels of involvement in the assignment. Faced with this situation, the teachers wondered whether they should intervene in problem solving among students or let the students work it out by themselves. An example of this is: “On several occasions I have found that in certain work groups, days before the assignment is due, they tell me that someone in the group hasn’t done their part or that they want to hand in the assignment leaving out a person from the group who has not been involved.”

Excess content in the curricula. The teachers highlighted the need to reduce the number of topics to be covered in the teaching plan. Teachers commented that they perceive developing active and constructive learning strategies such as problem-based learning as incompatible with completing the entire planned curriculum. The following is a teacher’s verbalization: “I’d love to use active techniques and encourage game-based learning, but I have specific content to teach, and I have to cover the entire curriculum. If I develop active techniques, I can’t complete the lesson plan.”

Student motivation and cohesion processes. Teachers stressed that one of the main difficulties encountered on a daily basis is motivating students. In this case, teachers identified two specific needs. First, they noted the need to have specific teaching skills or techniques to enhance student motivation. Second, the teachers revealed that sometimes they find themselves with groups that are not very cohesive, and that this makes the teaching-learning processes more difficult. Therefore, they need specific strategies or specific dynamics to achieve group cohesion. Here, teachers noted the importance of the initial classes and the need to develop specific teaching skills to establish a close bond between group members. The following is one teacher’s comment: “I worry about students who stop coming to class because they are not motivated. I wonder what I can do? In addition, sometimes there are also small groups of students who come and do not participate or get involved with the rest of the group.”

Managing oppositional student behavior towards the teacher. Although to a lesser extent than the other topics presented, teachers voiced concern about increasing oppositional or confrontational student behavior towards teachers. Some teachers noted that they find themselves lacking teaching skills to manage conflict with students or that they would not know how to react in the event this were to occur. Other teachers also expressed their concern that a student might react aggressively either in class or in tutoring. One teacher stated: “I have had a student question my authority. And at that moment, I was afraid of the student’s impulsive reaction and I was paralyzed.” 

### 3.2. Effectiveness of Psychodramatic Techniques in the University Setting

**Figure 3:**
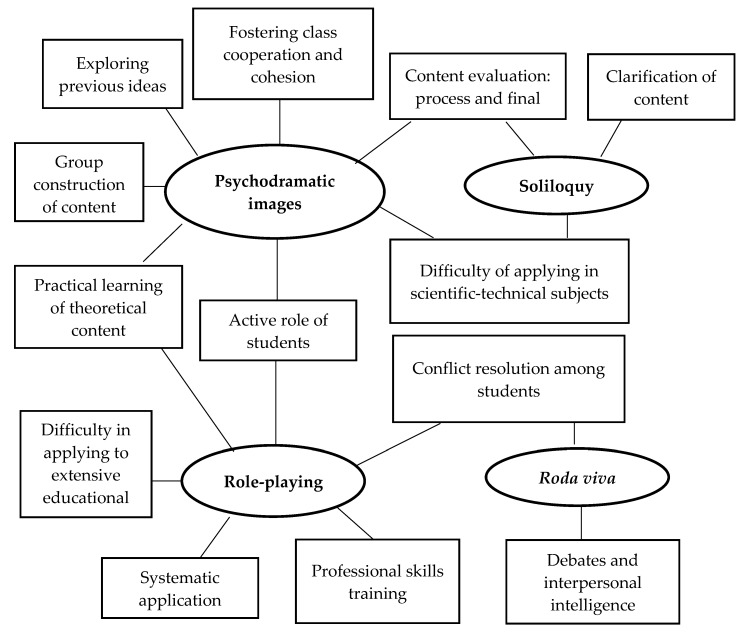
Perceived effectiveness of psychodramatic techniques.

Regarding the second objective, the themes identified in at least one third of the focus groups for each psychodramatic technique are shown below. Figure 3 illustrates the teachers’ assessment of the perceived usefulness, strengths and disadvantages of psychodramatic techniques in higher education.

Psychodramatic images. This was the technique most highly valued by teachers after TTS-EP. Teachers positively valued the usefulness of this technique to dynamically explore students’ previous ideas as a strategy for constructing new academic content in a meaningful way. In addition, teachers revealed that this technique can be used as a tool to evaluate the level of knowledge acquired by students during the teaching-learning process. Teachers also noted the usefulness of the technique to encourage shared construction of content among students. This technique also promotes group cohesion as students focus on the same activity, contributing different elements. Teachers described this technique as an opportunity to transform theoretical constructs into analogical images and to exemplify and deepen different characteristics of the curriculum. Teachers also noted its potential to promote active learning, which involves students in transforming a theoretical content into an image through cognitive processing of a specific theme. In contrast, teachers mentioned the difficulty of applying psychodramatic images in more abstract content typical of Science and Engineering, its application being more useful in areas such as Education, Psychology or History. The following is a comment by a teacher in which some of the identified themes appear: “I believe that psychodramatic images are a useful resource for students to get involved jointly and actively, while at the same time it is a challenge for them to transform a theoretical content into an image. I also consider that it can be a new useful tool to evaluate student learning.”

Soliloquy. Teachers commented on the importance of soliloquy to deepen the content incorporated in the psychodramatic image. In this case, teachers viewed soliloquy as an opportunity to clarify and define elements in the psychodramatic images, while at the same time it can be a tool for evaluation processes. Just as with psychodramatic images, teachers emphasized the difficulty of applying soliloquy in quantitative areas. The following is from a teacher after TTS-EP: “In my opinion, the soliloquies complete the image. For example, in the explanation of cells, I see it as an opportunity for the student to create the image of the cell and then define each component, how it changes or how it is transformed. It allows me to discover what the student has understood, and what I must reinforce as a teacher.”

Role-playing. This technique was positively valued by teachers. Although teachers mentioned that they already regularly used this technique in their classes, they valued the structure and the variants of the techniques proposed in TTS-EP. They also agreed that role-playing promotes an active teaching-learning process and increases the emotional component of learning. They also valued the opportunity that role-playing offers to practice the professional skills and competences that students will need to develop in their field. Thus, the teachers noted the importance of techniques to work on the professional skills of undergraduate students studying Psychology, Education, Law, Pharmacy, Nursing or Medicine. Additionally, teachers valued the importance of the different variants of role-playing. Specifically, the role-playing techniques known to students, spontaneous role-playing and fragmented role-playing, were valued for their usefulness in training students in their professional role. Teachers remarked that when a part of the dramatization is unknown to one of the students or to the class group, attention and motivation processes may increase due to the uncertainty of the situation to be dramatized. Similarly, the teachers valued the importance of planned role-playing to promote students’ cognitive processing of content and their subsequent transfer to practical situations. As a negative aspect, teachers revealed the considerable amount of time needed for role-playing and that this technique can, therefore, be incompatible with extensive curricula. One teacher’s comment on the usefulness of role-playing is: “TTS-EP has helped me to structure the use of role-playing. It will allow me to use it more systematically, taking into account the degree of information processing I want from the students before the dramatization. I also think it is an essential technique for improving professional skills; students like it and have fun.”

*Roda viva*. The *roda viva* was positively valued by the teachers in two educational applications. First, it can be used to conduct debates where students have to adopt different ideas. The teachers noted that this technique can foster different reasoning processes, as well as promote interpersonal intelligence by having students assume different positions during debates. They also expressed the usefulness of this technique in private tutoring to resolve possible conflicts in small groups of students. Secondly, the teachers mentioned that the *roda viva* is a resource for students to resolve their own intra-group conflicts by encouraging the adoption of the perspectives of different group members. According to the teachers, the *roda viva* can promote consensual solutions among students without the direct participation of the teacher. The following is a teacher’s view of *roda viva*: “What I will use most from the course is the *roda viva*. I believe it is a good technique to resolve conflicts between students without the teacher being responsible for the solution. In addition, now that the University is asking us to do so, we can work on transversal student skills such as conflict resolution strategies.”

## 4. Discussion

Research into evidence-based teaching practices is one of the main challenges facing universities in the 21st century. The study of this topic must be guided by a rigorous scientific process that will improve teaching-learning processes [7,8]. In learning processes, two main agents can be distinguished: the teacher and the student. Thus, the proposal of new teaching strategies must be integrated with prior reflection and assessment of the current needs of university teachers. To do this, teachers must be given a voice, and new teaching practices must be developed based on their expressed needs [1,8]. The results of this study have provided a map of the needs described by university teachers participating in the TTS-EP course. The application of educational psychodrama in the university setting has been presented, and initial evidence of the usefulness of psychodramatic techniques for teaching and learning processes has been obtained.

Concerning the first objective of the study, the inductive discourse analysis identified six needs. According to the theoretical model of teaching effectiveness by Medley [14], we can distinguish three teaching needs related to teaching style and three needs related to managing a group of students.

First, regarding teaching practices at the University, teachers indicated the impossibility of carrying out active practices with large classes of students, as well as the difficulty of finding teaching strategies to motivate students. These teaching needs are consistent with the main challenges shown by teaching approaches, such as constructionism that seeks meaningful learning based on student needs, and active and experiential learning [4,5,32]. The meaningful learning appears to be enhanced when teachers are aware of the previous ideas of the students, their needs and when they connect academic content to the experiences, emotions and motivations of the students [5,16]. Similarly, teachers revealed the need to acquire active teaching strategies to increase student motivation, consistent with current studies that report a strong association between student motivation and academic performance [10]. This is also in accordance with current learning approaches such as flipped room, problem-based learning or project-based learning. These evidence-based approaches are underpinned by the importance of basing teaching-learning processes in active and practical situations for students [6,11].

Teachers participating in TTS-EP indicated that they need to improve their skills and strategies in the early stages of teaching in order to improve group cohesion. This detected need to implement measures for group cohesion as a basic process to facilitate learning is consistent with different theories of group processes [48,49]. According to these theories, the first phase of the group may be defined by an internal chaos that makes it difficult for the group members to perform. Teachers must acquire strategies to facilitate progression from this initial chaos (students do not know the goals of the course, do not know the expected behaviors in the class and do not know their other classmates) to a phase of group identity or performance (students know the formal aspects of the subject, the rules of the class and their role in the class group in relation to other students) to improve the results of the teaching-learning process [11]. University teacher training courses should, therefore, recognize teachers’ current needs for guidance and educational tools to develop active teaching practices. This would facilitate teaching-learning processes in large groups and help unite and motivate students.

Second, according to predictive dimensions of teacher effectiveness [14], teachers revealed needs associated with the acquisition of skills to manage groups of students. The first need focused on the distance in student-teacher interactions. Although there were discrepancies between teachers, most indicated that they based their teaching performance on close and warm relationships with the student. Nevertheless, teachers noted their fear of losing their authority should they position themselves as partners in the student’s learning. Possibly, certain beliefs rooted in the traditional educational model based on the distance between teacher and student and on the presence of hierarchy may justify the fear among these teachers of maintaining a closer relationship with students. However, previous studies show that students positively value having warm and close teachers [50,51].

Among the most salient results is the need for a range of strategies to respond to conflicts in higher education. Specifically, teachers highlighted two types of conflicts: conflicts between students and conflicts between teacher and student caused by oppositional behaviors and student confrontation with the teacher. Although these types of problematic situations in the classroom have been widely addressed in primary and secondary education, less data are available on oppositional behaviors in the university setting [13]. Higher education has placed greater emphasis on the development of protocols to prevent violent behavior among students, such as the prevention of gender violence and harassment [52]. Therefore, it is a challenge for teachers to develop conflict resolution strategies in universities today. This need should be addressed in the various teacher training plans proposed by different universities. 

Concerning the second objective, on a conceptual level, this study aims to be consistent with the current state of teaching effectiveness, which reveals that teaching excellence can only be achieved through research-informed pedagogical practices [3]. Additionally, current studies confirm that teachers must be trained in practices that encourage good student performance [11]. According to this line of research, the application of educational psychodrama in the university setting has been described in this paper. Additionally, a forum has been created for teachers to assess the usefulness of psychodramatic techniques. Although educational psychodrama has been proposed as a teaching method for decades and recent experiences in higher education exist [29,38,39], a lack of knowledge persists regarding the evidence and effectiveness of these techniques. Overall, teachers positively evaluated psychodramatic techniques to foster active, experiential and motivational learning and as resources for learning assessment and class group management. Similarly, from an integrative approach, psychodramatic techniques can become a teaching resource to address some of the needs raised by teachers in the first phase of the TTS-EP course.

The first implication of the results on the usefulness of psychodramatic techniques was the relevance of psychodramatic images. These techniques encourage active learning in large groups and cohesion in class groups as well as improving formal aspects of evaluation: exploration of previous ideas, process evaluation or final evaluation. Specifically, this study provides initial evidence of the usefulness of psychodramatic images as a technique in the university setting. This technique has traditionally been used in clinical and social contexts; its educational application in secondary education is limited to adolescents having reached a certain level of formal reasoning [28,35]. To the extent that the psychodramatic image facilitates both practical and formal aspects of evaluation in higher education, the perceived usefulness of this technique is justified. In addition, teachers participating in TTS-EP mentioned the importance of achieving meaningful learning by students. According to the constructionist approach [5], psychodramatic images can enhance different processes such as: (a) the exploration of students’ previous ideas; (b) the relationship between new content and content already acquired by students; (c) experiential learning; and (d) active and cooperative participation.

The findings regarding psychodramatic images are complemented by the perceived effectiveness of the soliloquy in exploring and encouraging students to deepen and clarify the content developed in the psychodramatic images. The soliloquy has been revealed as one of the most useful techniques in psychodrama [24]. Therefore, this technique is suitable for use in higher education to foster learning processes through the cognitive and verbal elaboration of content. However, according to the recommendations of interdisciplinary learning [53], the knowledge of specific psychodramatic techniques should be extended to cover content specific to quantitative sciences as perceived by the teachers. In summary, psychodramatic images and soliloquy appear to respond to some of the needs initially raised by teachers and represent new tools to maximize positive effects in teaching-learning processes.

The second implication of the techniques was the perceived effectiveness of role-playing. Role-playing was considered useful to promote active learning and to facilitate the translation of theoretical content into practical aspects. The purpose of role-playing in education and the importance of developing kinesthetic learning have been frequently addressed in the literature [27,28,54]. Additionally, applications of educational psychodrama in higher education have demonstrated the usefulness of role-playing to develop professional skills in university students [29,38,39]. However, although teachers mentioned they already use this technique in teaching-learning processes, they highlighted the usefulness of the systematization and structuring proposed in the TTS-EP course. According to the diversity in student learning styles, such as students who tend towards active learning as opposed to students who tend towards reflective learning [32], teachers stressed the diversity of changing mechanisms for learning (cognitive processing, information processing, action, spontaneity and creativity) that support the different variants of role-playing presented. Teachers valued the efficiency of the dichotomy between planned role-playing that involves the reflection on and the cognitive preparation of content and other role-playing alternatives that promote creativity and spontaneity. According to the theoretical articulation of pedagogical role-playing and to the results found on its use at universities [29,38], training students’ spontaneity and creativity can improve their professional skills. Previously, role-playing had been shown to be effective with psychology and business students [29,39]. In this way, participation in TTS-EP by teachers from different disciplines can help systematize the use of role-playing as a transversal technique for the training of professional skills in different fields.

Finally, the third implication of psychodramatic techniques is the effectiveness of the *roda viva*. This technique is perceived as useful for engaging in debates and for improving students’ personal skills such as interpersonal intelligence for conflict resolution. This promotion of the adoption of perspective occurs when the student defends all the opinions in the debate; first, the student defends opinion a, then turns and defends opinion b and so on. Currently, the teaching plans state the importance of working on students’ transversal skills [55]. Traditionally, psychodramatic techniques have shown value in promoting personal skills such as empathy, conflict resolution strategies or self-knowledge [24,25,56]. According to the teachers, educational use of the *roda viva* can improve personal skills and, consequently, can be beneficial to students’ future careers.

In summary, consistent with previous studies in higher education [29,38], teachers positively valued the use of psychodramatic techniques to enhance learning. Despite the positive effects of psychodrama on social and psychotherapeutic intervention [25], its application in higher education should be limited to the learning process and the pedagogical content. To respect ethical principles and according to authors promoting educational psychodrama [29], psychodramatic techniques should focus on the acquisition of knowledge and professional training rather than on personal events of students and teachers.

This study has several limitations. First, the participants may be highly motivated to improve and refine their teaching skills, which can create purposive sampling bias. It would, therefore, be helpful to determine whether the needs expressed concerning the teaching-learning processes would be similar in teachers who do not participate in teaching innovation courses. Second, it would have been advisable to collect other sociodemographic data such as the age of the teachers or data such as the motivation for attending training. Third, although a rigorous qualitative analysis was undertaken, it would be of interest to use standardized questionnaires to quantitatively validate the needs expressed by teachers from a randomized sample at other universities. Fourth, differences according to teaching experience have not been explored. This variable can mark the perception of different needs [14]. Fifth, it is advisable to monitor the application of psychodramatic techniques in the respective disciplines of each teacher. Finally, the psychodramatic techniques are presented in a general and interdisciplinary way [50]. The usefulness of the techniques could be improved by making connections between specific applications of the techniques and the academic curriculum.

## 5. Conclusions

This study provides initial evidence of the usefulness of educational psychodrama in higher education. The results have a number of practical implications for improving teaching and university teacher training programs. Specifically, these implications arise from the interdisciplinary and uninterrupted implementation of TTS-EP over a period of three years.

Teachers have been given a voice to reflect upon and express their opinion on current educational needs. This study has two main implications. The first is the need for teachers to improve educational skills to promote active, meaningful and motivating learning, especially in large groups [4]. The second is the finding that conflicts are increasing in small work groups and in the teacher-student relationship. Teachers must develop effective strategies to resolve these conflicts. Accordingly, these teaching needs should be addressed in university teacher training programs [7,8].

Finally, with regard to educational psychodrama, psychodramatic images are revealed as a very effective technique for achieving active and cooperative learning, motivating a group and translating theoretical content to practical content. In addition, psychodrama can be a resource for exploring previous ideas and for evaluating learning. Teachers highlighted the systematic application of role-playing variants to promote different learning processes. TTS-EP provides teachers with a series of practical resources that can complement and be integrated into other current models such as problem-based learning or project-based learning [21,22]. In short, this study provides empirical support for the use of psychodramatic techniques as a resource to promote excellence in teaching-learning processes and positive student outcomes.

## Figures and Tables

**Table 1:** Educational application of psychodramatic techniques.

Techniques	Definition	Variants
Psychodramatic images	Construction of a figure with the rest of the students that analogically represents the academic contents	a. Image developed by the teacher b. Image developed by one or two students c. Image developed by small groups on similar content d. Image developed by small groups on different related content e. Progressive image developed by all the students
Soliloquy	Development of a discourse on the different academic contents of the image. This discourse is created by the same student or group that has constructed the image	
Role-playing	Dramatization by at least two students of an academic content.	a. Planned role-playing (content known—content known to both students) b. Role-playing known to one student (content known by one student—content unknown to the other student) c. Spontaneous role-playing (content unknown—content unknown to both students) d. Fragmented role-playing (content known only to the students performing the role-playing or only to the rest of the class group)
*Roda viva*	Rotating and adopting different roles regarding ideas, thoughts or content proposed by different students	
